# Individual dental and skeletal age assessment according to Demirjian and Baccetti: Updated norm values for Central-European patients

**DOI:** 10.1007/s00056-022-00431-5

**Published:** 2022-10-14

**Authors:** Eva Paddenberg, Adrian Dees, Peter Proff, Christian Kirschneck

**Affiliations:** Department of Orthodontics, University Hospital Regensburg, Franz-Josef-Strauß-Allee 11, 93053 Regensburg, Germany

**Keywords:** Cervical vertebral maturation stages, Orthopantomograms, Lateral cephalograms, Individualized diagnostics, Tooth mineralisation stages, Skelettales Alter, Dentales Alter, Norm-Werte, Individualisierte Diagnostik, Zahnmineralisierungsstadien

## Abstract

**Purpose:**

Chronological age often differs from dental and skeletal age. With orthopantomograms and lateral cephalograms, dental and skeletal development can be determined according to the methods published by Demirjian et al. and Baccetti et al. However, gender and skeletal class as possible confounders were frequently not considered and available norm values are not up-to-date. This retrospective cross-sectional study thus aimed to evaluate effects of skeletal class and gender on dental and skeletal age of growing patients and to generate updated norm values for contemporary Central-European patients.

**Methods:**

A total of 551 patients were included in the dental and 733 in the skeletal age assessment, respectively. Dental analysis was based on tooth mineralisation stages in orthopantomograms (Demirjian) and skeletal age was defined by cervical vertebrae maturation stages (CVMS) in lateral cephalograms (Baccetti). Skeletal class was determined by the individualised ANB angle of Panagiotidis/Witt. With nonlinear regression analysis a formula for determining dental age was established. Effects of gender and skeletal class were evaluated and updated norm values generated.

**Results:**

Inter- and intrarater reliability tests revealed at least substantial measurement concordance for tooth mineralisation and CVMS. Demirjian stages and CVMS significantly depended on gender with girls developing earlier. Skeletal class significantly affected skeletal age only, but without clinical relevance. Updated norm values for dental age differed significantly from the original values of Demirjian and the values for skeletal age differed from those published by Baccetti.

**Conclusion:**

Optimised norms, separated by gender, increase precision in determining individual dental and skeletal age during orthodontic treatment planning. Further studies analysing the effect of skeletal class on dental and skeletal development are needed.

## Abstract (de)

**Zweck:**

Das chronologische Alter weicht oft vom dentalen und skelettalen Alter ab. Mit Orthopantomogrammen und Fernröntgenseitenbildern kann die dentale und skelettale Entwicklung nach den von Demirjian et al. und Baccetti et al. publizierten Methoden bestimmt werden. Allerdings wurden Geschlecht und skelettale Klasse als mögliche Kovariablen häufig nicht berücksichtigt, und verfügbare Normwerte sind nicht aktuell. Ziel dieser retrospektiven Querschnittsstudie war es daher, die Auswirkungen der skelettalen Klasse und des Geschlechts auf das dentale und skelettale Alter heranwachsender Patienten zu evaluieren und aktualisierte Normwerte für zeitgenössische mitteleuropäische Patienten zu generieren.

**Methoden:**

Insgesamt 551 Patienten wurden in die Beurteilung des dentalen Alters, 733 Patienten in die des skelettalen Alters eingeschlossen. Die dentale Analyse basierte auf Zahnmineralisierungsstadien in Orthopantomogrammen (Demirjian), und das skelettale Alter wurde durch Reifestadien der Halswirbel (CVMS) in Fernröntgenseitenbildern (Baccetti) definiert. Die skelettale Klasse wurde durch den individualisierten ANB-Winkel von Panagiotidis/Witt bestimmt. Mittels nichtlinearer Regressionsanalyse wurde eine Formel zur Bestimmung des dentalen Alters generiert. Effekte von Geschlecht und skelettaler Klasse wurden ausgewertet und aktualisierte Normwerte generiert.

**Ergebnisse:**

Überprüfungen von Inter- und Intrarater-Reliabilität ergaben zumindest eine gute Übereinstimmung der Messungen für die Mineralisierungsstadien und CVMS. Demirjian-Stadien und CVMS hingen signifikant vom Geschlecht ab, Mädchen entwickelten sich früher. Die skelettale Klasse beeinflusste nur das skelettale Alter signifikant, jedoch ohne klinische Relevanz. Die aktualisierten Normwerte für das dentale Alter wichen deutlich ab von den ursprünglichen Werten von Demirjian, die Werte für das skelettale Alter unterschieden sich von den von Baccetti veröffentlichten Werten.

**Schlussfolgerungen:**

Optimierte Normen, getrennt nach Geschlecht, erhöhen die Präzision bei der Bestimmung des individuellen dentalen und skelettalen Alters bei der kieferorthopädischen Behandlungsplanung. Weitere Studien zur Analyse der Auswirkungen der skelettalen Klasse auf die dentale und skelettale Entwicklung sind erforderlich.

## Introduction

Orthodontic decision-making requires knowledge about dental and skeletal development to optimise timing of orthodontic treatment in dependence of biological age [51]. Skeletal maturity provides information about expected growth potential, which is necessary for orthopaedic effects, and dental age acts as a first (clinical) evaluation of an individual’s developmental stage during initial diagnostics.

Several methods exist to determine an individual’s biological age [29] as opposed to the patient’s actual chronological age, among which radiographic imaging is a major contributing factor for dental and skeletal age assessment. Whereas specific radiologic imaging such as hand–wrist radiographs result in additional radiation and require strict indications, orthopantomograms and lateral cephalograms are readily available as part of routine diagnostics throughout orthodontic treatment.

In 1972, Lamparski [31] determined skeletal maturity in lateral cephalograms by evaluating changes in shape, size and ratios of cervical vertebrae and distinguished between six grades, which generally occurred earlier in girls than in boys. This idea was further developed by Hassel and Farman [20], who established six cervical vertebral maturation (CVM) indicators, based on growth-related changes in C2, C3 and C4 which were associated with growth potential, as a comparison with hand–wrist radiographs showed. Baccetti et al. [4] optimised this technique by analysing the same cervical vertebrae in six consecutive lateral cephalograms of 30 patients and published five CVM stages (CVMS). Although in literature contradictory information exists, several studies concluded that lateral cephalograms are often equally suitable as hand–wrist radiographs to determine skeletal age [18, 37, 46].

Teeth were found to erupt within certain chronological time intervals, enabling the clinician to assess dental age by comparing the individual tooth status. However, as there is a range of 2 years in forecasting tooth eruption [43], the clinical prediction of dental age is imprecise und not appropriate to establish a treatment plan. In the 1940s, Schour and Massler [53, 54] discovered the continuity of dental development. Their atlas method, a comparison between schematic graphs and individual dental developmental stages in orthopantomograms [54], is still used today, although other methods also exist to assess dental age [40, 52]. Demirjian et al. [12] analysed mineralisation stages of teeth in the third quadrant using orthopantomograms to define dental age, separated by gender.

Sagittal dysgnathias are subdivided into three groups: normal relationship between the upper and lower jaw (skeletal class I), distobasal jaw relation (skeletal class II) and mesiobasal jaw relation (skeletal class III). A patient’s skeletal class can be determined by several parameters in cephalometric analysis, but floating norms, considering vertical and sagittal variables and thus the influence of vertical jaw development on sagittal changes, proved to be reliable and valid [55]. One method used to individualise the ANB angle is the regression equation of Panagiotidis and Witt [45].

In the literature contradictory information about a possible association between the skeletal class and dental/skeletal age has been published. Although some authors [9] found a correlation between dental age and sagittal dysgnathia, others [24] described a relationship between dental age and vertical dysgnathia, which was not confirmed by Jamroz et al. [23]. Morphology of the cervical vertebrae, used as an indicator of skeletal development, seems to be influenced by skeletal class in adult patients [58]. A recently published study reported skeletal class- and gender-related differences in skeletal age, although the clinical relevance was rather small [48]. The methods of Demirjian et al. and Baccetti et al. to determine dental and skeletal age, respectively, are generally accepted, but there are some limitations. Furthermore previous studies have questioned the reliability of the techniques of Demirjian et al. [19] and Baccetti et al. [17]. Both are based on a population different from contemporary Central-European patients and do not consider skeletal class as a possible confounding variable. Furthermore, the CVMS method of Baccetti et al. did not investigate gender-related differences in skeletal maturation and relied on a small sample (*n* = 30).

Therefore, the aim of this study was primarily to assess the association between dental and skeletal age, using the methods of Demirjian [12] and Baccetti [4], respectively, on the one hand, and gender and skeletal class on the other hand in growing orthodontic patients. Secondly, updated norm values for dental and skeletal age for a contemporary Central-European population should be generated.

## Materials and methods

In this retrospective cross-sectional study orthopantomograms and lateral cephalograms of 400 consecutively selected patients per skeletal class (I, II and III, *n* = 1200 in total) were screened for eligibility. Due to the multicentric study design, patients were recruited at two different specialist orthodontic practices in Germany (Bavaria and Baden-Wuerttemberg). Only those radiographs taken for diagnostic purposes between 1993 and 2013 were considered. For each patient and time (before, during or after treatment) gender and chronological age were recorded. Ethnicity, a potential confounder, was not documented, since comparable ethnic groups were assumed due to the exclusively German study locations. Orthopantomograms were used to assess dental age, whereas lateral cephalograms were evaluated for the determination of skeletal age and skeletal class. The study was performed in concordance with the Declaration of Helsinki (2013), the ethical guidelines of the University of Regensburg (approval of the ethical committee: 19-1596-101) and the Strengthening the Reporting of Observational studies in Epidemiology (STROBE) guideline.

**Fig. 1:**
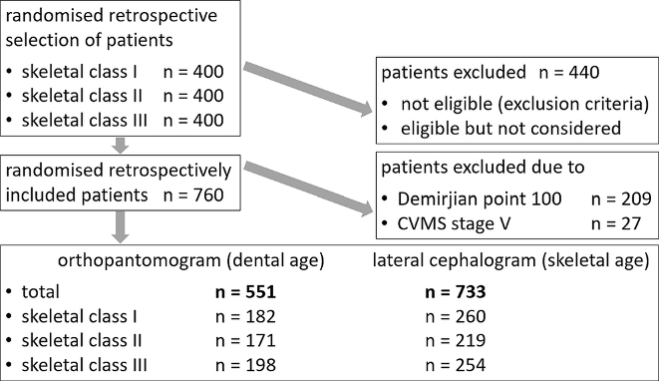
Flowchart of study collective. *CVMS*: cervical vertebrae maturation stages Flowchart des Studienkollektivs. *CVMS*: „Reifestadien der Halswirbel“

To avoid distortion of the data, patients were not eligible for this study if systemic, local, exogenous, iatrogenic or genetical factors could have affected dental or skeletal development or if this maturation was not clearly assessable. Moreover, no patients younger than 6 or older than 20 years were considered because radiographs at these chronological ages were scarce and would not allow meaningful statistics. Also those patients who did not have a lateral cephalogram and orthopantomogram taken within 3 months at initial, interim or final examination were excluded to enable statistical testing of both dental and skeletal parameters. Finally, poor quality radiographs, hindering analysis of the predefined outcomes (mineralisation stage, CVMS, individualised ANB, skeletal class) led to exclusion. Patients with 100 Demirjian points (meaning completion of root development) and CVMS V were excluded from further analyses, since these stages are not assigned to a specific dental/skeletal age and could introduce bias into the statistics. Finally, 551 patients (265 male, 286 female) were recruited for dental age assessment and 733 patients (374 male, 359 female) for determination of skeletal maturation (Fig. 1). All participants were anonymised and randomised directly at the source. For further analyses, patients were subdivided in four chronological age groups (≤ 10 years, 10–12 years, 12–14 years, > 14 years).

Dental and skeletal age were analysed by one rater (AD) under blinding of the chronological age using the orthopantomograms and lateral cephalograms at a randomised time, i.e. initial, interim or final examination. Cephalometric analysis covered only measurement of the individualised ANB angle of Panagiotidis and Witt [45] to determine the skeletal class. If analysis could not be performed at the randomly chosen timepoint, another date was randomly selected.

Within the time interval from 1993–2013 the radiographic devices were switched from analogue to digital in both orthodontic offices. Analogue images were taken with the device OrthOralix FD ceph (Philips Gendex Dental-Systeme, Hamburg, Germany) and digital radiographs with the device Veraviewepocs 2D (J. Morita Europe GmbH, Dietzenbach, Germany). The following settings were applied for the orthopantomograms: anode voltage of 63–69 and 62–69 kV, anode current strength of 6.0 and 5.3–7.3 mA and an exposure time of 17 and 14.8 s in analogue and digital images, respectively. Lateral cephalograms were conducted with an anode voltage of 69–73 and 70–80 kV, an anode current strength of 6.0 and 7.0–7.2 mA and an exposure time of 12 and 4.9 s in analogue and digital radiographs, respectively.

Orthopantomograms (outcome: Demirjian point value) and lateral cephalograms (outcome: CVMS) were analysed using an x-ray viewer (Rex Messinstrumentenbau GmbH, Erlangen, Germany) and an approved monitor MDview21 (NEC, Munich, Germany) for analogue and digital images, respectively. Analogue lateral cephalograms (skeletal class) were plotted manually with acetate overlay, digitised with the digitiser Summa Sketch II Plus, model MM II 1201 (Summagraphics, Seymour, CT, USA), and cephalometrically analysed using the Fernröntgendiagnose software (version 1.6; Dr. Klaus Keß, Wuerzburg, Germany). Digital lateral cephalograms (skeletal class) were evaluated with the program FRS4KFO version 5.0.0.d of the KFOOFFICE software (FDK-Fachdienst der Kieferorthopäden, Nienburg, Germany).

**Fig. 2:**
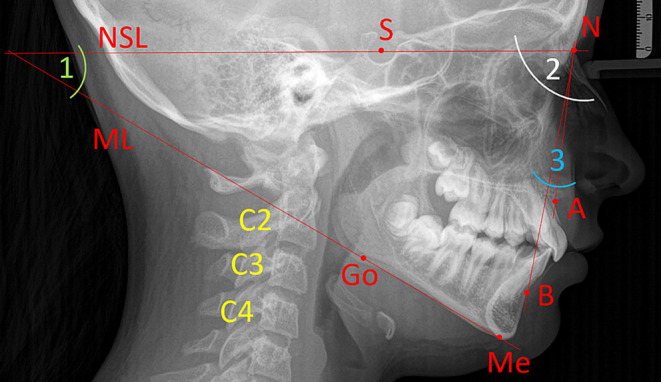
Cephalometric analysis to analyse skeletal class according to the individualised ANB angle by Panagiotidis and Witt [45] and maturation stages of cervical vertebrae C2–C4. *N* nasion, *S* sella, *A* point A, *B* point B, *Me* menton, *Go* gonion, *ML* mandibular line, *NSL* nasion-sella line, *angle* *1* *ML-NSL* inclination of the mandible, *angle* *2* *SNA* maxillary prognathism, *angle* *3* *ANB* sagittal relation between upper and lower jaw (skeletal class) FRS(Fernröntgenseitenbild)-Analyse zur Bestimmung der skelettalen Klasse anhand des individualisierten ANB-Winkels von Panagiotidis und Witt [45] sowie der Reifestadien der Halswirbel C2–C4. *N* Nasion, *S* Sella, *A* A-Punkt, *B* B-Punkt, *Me* Menton, *Go* Gonion, *ML* Mandibularlinie, *NSL* Nasion-Sella-Linie, *Winkel* *1* *ML-NSL* Inklination des Unterkiefers, *Winkel* *2* *SNA* maxillärer Prognathiegrad, *Winkel* *3* *ANB* sagittale Relation zwischen Ober- und Unterkiefer (skelettale Klasse)

Skeletal class was defined by comparing measured and individualised ANB angle of Panagiotidis and Witt [45], which was calculated applying the following formula (Fig. 2):

1 $$\textit{Indiv.ANB angle}_{\textit{Panagiotidis and Witt}}=-35.16+0.4\times SNA+0.2\times ML-NSL$$

Skeletal class II was diagnosed if the measured ANB exceeded the individualised ANB by at least 1 °; skeletal class III was determined if the measured ANB was at least 1 ° smaller than the individualised ANB. A difference within a range of 1 ° in either direction resulted in the diagnosis of a skeletal class I.

The CVMS method of Baccetti et al. [4] was used to define skeletal age: cervical vertebrae C2, C3 and C4 were evaluated in a single lateral cephalogram visually and classified into one of five CVMS (Table 1, Fig. 2). Not absolute sizes, but ratios of the distances in cervical vertebrae were important. In contrast to the original method, we distinguished between male and female patients.

**Table 1:** Cervical vertebrae maturation stages (CVMS) I–V to assess skeletal age with lateral cephalograms. (Technique modified after Baccetti et al. [4]) CVMS I–V („Reifestadien der Halswirbel“) zur Ermittlung des skelettalen Alters anhand von FRS(Fernröntgenseitenbild)-Aufnahmen. (Methodik modifiziert nach Baccetti et al. [4])

CVMS	Figure	Definition	Mandibular growth potential
CVMS I	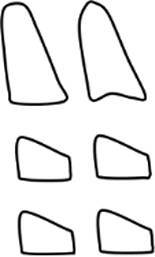	Lower borders of C2–C4 are flat. C2 may have a concavity. C3 and C4 are trapezoidal and conical from posterior to anterior	Growth peak occurs at least one year after this stage
CVMS II	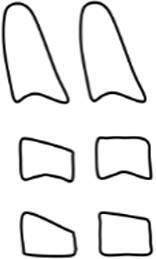	Lower borders of C2 and C3 are concave. C3 and C4 are trapezoidal or horizontally rectangular	Growth peak occurs within half a year after this stage
CVMS III	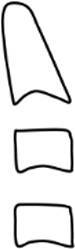	Lower borders of C2, C3 and C4 are concave. C3 and C4 are horizontally rectangular	Growth peak has started one or two years before this stage
CVMS IV	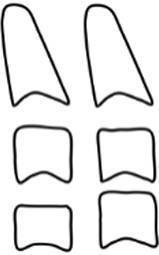	Lower borders of C2, C3 and C4 are concave. At least one of C3 and C4 is square, otherwise the other is horizontally rectangular	Growth peak has started within one year before this stage
CVMS V	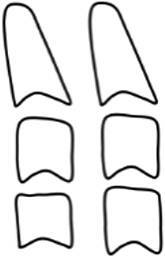	Lower borders of C2, C3 and C4 are concave. At least one of C3 and C4 is vertically rectangular, otherwise the other is square	Growth peak has started within two years before this stage

**Fig. 3:**
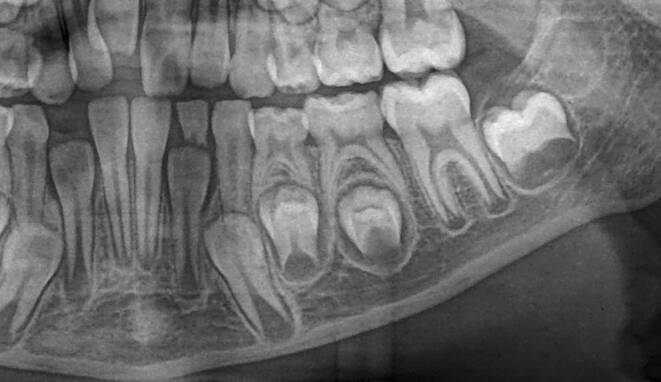
Orthopantomogram section of the lower left quadrant with different Demirjian stages (31: H, 32: F, 33: F, 34: D, 35: C, 36: G, 37: C) Orthopantomogramm-Ausschnitt des linken unteren Quadranten mit verschiedenen Demirjian-Stadien (31: H, 32: F, 33: F, 34: D, 35: C, 36: G, 37: C)

Dental age was assessed using the method of Demirjian et al. [12]: orthopantomograms were analysed to distinguish eight mineralisation stages (A–H) of teeth 31 to 37 (Table 2, Fig. 3). All teeth were equally significant and every stage was associated with a certain Demirjian point value, ranging from 0 to 100. The summarised point values of all teeth yielded in an individualised dental age. These were compared to correlation tables that were separated by gender. Evaluating mineralisation stages, no absolute measurements were conducted, but ratios and visual inspection were solely performed. Crown height was defined as the greatest difference between cusp tip and cementoenamel junction (CEJ) and in case of different heights the mean was calculated. In borderline cases the previous stage was chosen. If no calcification was visible, the tooth was given stage 0, corresponding to a Demirjian point value of 0. When teeth were missing, a summarised point value was calculated without considering the missing tooth/teeth.

**Table 2:** Eight mineralisation stages (A–H) to assess dental age and mandibular growth potential with orthopantomograms. (Method modified after Demirjian [12]) Acht Mineralisationsstadien (A–H) zur Bestimmung des dentalen Alters und des mandibulären Wachstumspotenzials anhand von Orthopantomogrammen. (Methodik modifiziert nach Demirjian [12])

Mineralisation stage			Figure	Definition
A			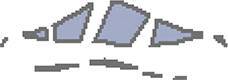	At single- and multiroot teeth calcification starts in the occlusal part as single or several points. Calcificated points are not merged
B			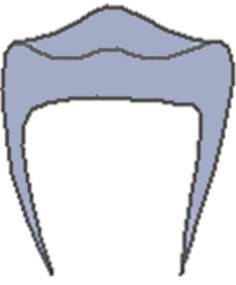	Fusion of calcificated points results in a consistent occlusal area and in the formation of one or more cusps
C		a	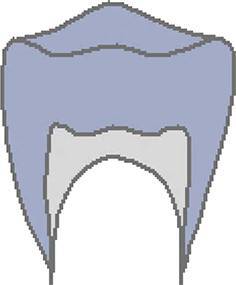	Development of enamel is terminated in the occlusal part. Extension and convergence occur in cervical direction
		b		Dentine deposit begins
		c		Dentine deposit leads to contouring of the top of the pulp cavity
D		a	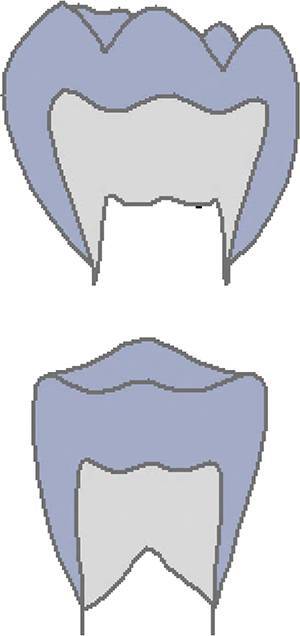	Crown formation is finished up to cementoenamel junction
		b		Contouring of the coronal part is defined in single-root teeth and concave in cervical direction. If pulp horns exist, they look like an umbrella or trapezium in single- and multiroot teeth, respectively
		c		Root formation starts and is visible as a needle-like process
E	Single-root teeth	a	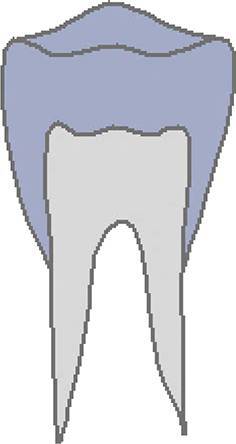	Root walls are straight and pulp horn is larger than in the previous stage
		b		Root length is still shorter than crown length
	Molars	a	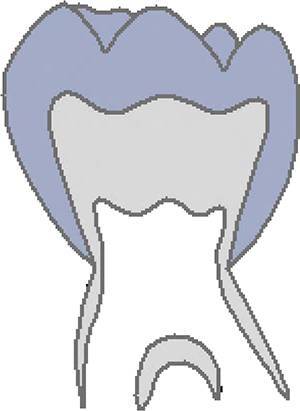	Formation of bifurcation starts in the form of a calcifying point or crescent
		b		Root length is still shorter than crown length
F	Single-root teeth	a	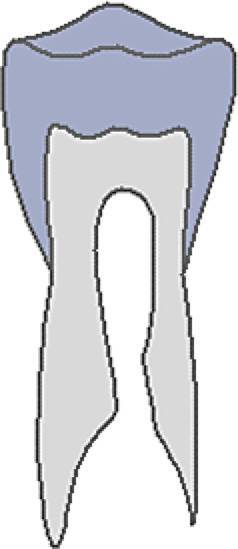	Shape of pulp chamber looks like a triangle. The opening of the apex looks chimney-like
		b		Root is as long as the crown or longer
	Molars	a	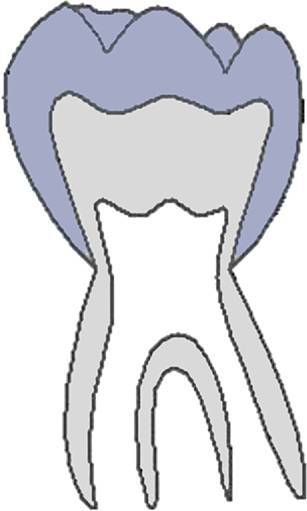	Clearly visible roots have developed from the crescent-shaped calcification of the bifurcation. The roots show a chimney-like opening
		b		Root is as long as the crown or longer
G			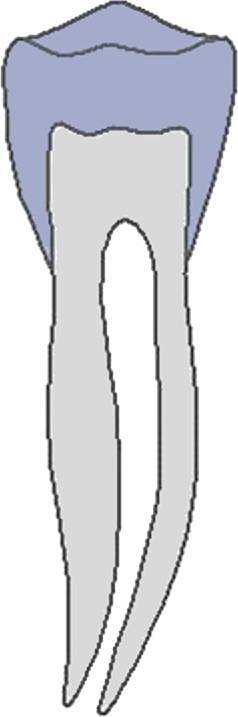	Root walls are parallel, but the apical portion is still partially open. At molars, this is especially true for the distal root
H			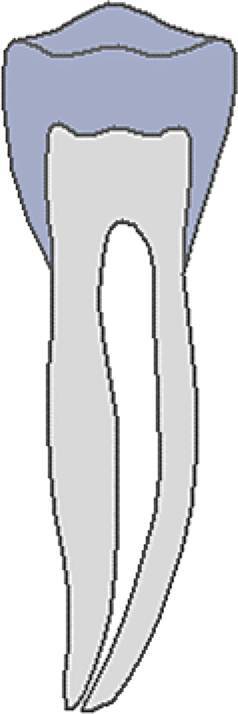	The apical tail of the root canal is completely formed and closed. At molars, this is especially true for the distal root. Periodontal gap is equally extended

Statistical analysis was performed using IBM® SPSS® Statistics 21 (IBM Deutschland GmbH, Ehningen, Germany) and the online accessible NIWA (NIWA Taihoro Nukurangi, 2013) for calculating Lin’s concordance correlation coefficient (CCC). Prior to the main investigation, 94 patients of all age groups were randomly selected for reliability testing. Blinding chronological age, lateral cephalograms and orthopantomograms were evaluated twice by the same rater (AD) with a time interval of at least 3 weeks and by a second rater to test intra- and interrater reliability, respectively. Lin’s CCC was applied for scalar-metric variables (Demirjian point value), Cohen’s kappa (κ) for categorical parameters (CVMS). The following interpretation, suggested by NIWA, was used: *p* > 0.99 perfect concordance, 0.95 < *p* ≤ 0.99 substantial, 0.90 ≤ *p* ≤ 0.95 moderate and *p* < 0.90 small concordance.

Normal distribution of the data was examined by Kolmogorov–Smirnov test and visual–optical inspection of histograms, variance homogeneity by Levene tests and visual–optical analysis of zpred vs. zresid plots. Analytical statistics for continuous normally distributed data included parametric independent two-tailed one-way analysis of variance (ANOVAs), corrected according to Welch in case of variance heterogeneity, with corresponding post hoc tests. If continuous data were not normally distributed, nonparametrical two-tailed independent Mann–Whitney or Kruskal–Wallis tests were performed. Categorical parameters were analysed with exact Fisher tests. Dependent two-tailed paired t‑tests and nonparametrical, dependent two-tailed Wilcoxon rank sum tests were applied to assess reliability of the analysis of dental age (Demirjian, Regensburger analysis) and skeletal age (Baccetti, Regensburger analysis) respectively. To establish a formula for dental age assessment, nonlinear regression analysis with a growth function (e-function) was applied, showing best fit according to the coefficient of determination R^2^. The analysis of skeletal age was based on conversion tables for CVMS and corresponding chronological ages, incorporating the results of descriptive and analytical statistics. Significance level was set at *p* ≤ 0.05, with corrections for multiple testing performed according to Bonferroni–Holm (BH). To judge clinical relevance of significant differences, Pearson’s correlation coefficient r and Cramer’s V were calculated as effect sizes for metrical and categorical variables, respectively; r or V > 0.5 represents a large, r or V > 0.3 a medium and r or V > 0.1 a small effect.

## Results

From 1200 inspected patients, 760, who equally contributed to skeletal classes I, II and III in each chronological age group, were finally included (Fig. 1, Table 3). Gender was equally distributed among all patients (49.6% male, 50.4% female) and the four age groups, showing no significant difference between male and female patients (*p* > 0.05).

**Table 3:** Distribution of skeletal classes (I, II, III) within the total study collective and the four age groups Verteilung der skelettalen Klassen (I, II, III) innerhalb des gesamten Studienkollektivs und der 4 Altersgruppen

Age group	Skeletal class I		Skeletal class II		Skeletal class III	
	*n*	%	*n*	%	*n*	%
Total	268	35.3	224	29.4	268	35.3
6–10	42	29.4	45	31.5	56	39.2
10–12	68	34.2	66	33.2	65	32.7
12–14	78	35.9	60	27.6	79	36.4
14–20	80	39.8	53	26.4	68	33.8

*n* absolute frequency of patients, *%* relative frequency within an age group

Inter- and intrarater reliability testing revealed at least substantial concordance for the determination of the Demirjian point value in the orthopantomograms and CVMS in the lateral cephalograms (κ > 0.8; ρ_c_ > 0.95).

Orthopantomograms of 551 patients were included in dental age assessment. Their distribution regarding age groups, mean values of chronological age, Demirjian point value and dental age after Demirjian is presented in Table 4. Demirjian point values of girls were significantly higher in all chronological age groups (*p* < 0.001), whereas no significant gender-related difference existed for dental age according to Demirjian in all chronological age groups except for the group > 14 years (*p* = 0.043/*p*^BH^ = 0.215). Dental age, as determined by Demirjian, was significantly higher than chronological age (*p* < 0.01/*p*^BH^ < 0.01) for all patients younger than 14 years (*p* = 0.098).

**Table 4:** Distribution of orthopantomograms (*n*), chronological age (in years), Demirjian point value and dental age according to Demirjian (in years) within the chronological age groups and the total study population Verteilung der Orthopantomogramme (*n*), des chronologischen Alters (in Jahren), des Demirjian-Punktwertes und des dentalen Alters nach Demirjian (in Jahren) innerhalb der chronologischen Altersgruppen und des gesamten Studienkollektivs

Age group	*n*	M ± SD (years)		
		Chronological age	Demirjian point value	Dental age
Total	551	11.3 ± 2.0	91.4 ± 9.6	11.7 ± 2.4
6–10	142	8.8 ± 0.9	80.8 ± 12.7	9.0 ± 1.4
10–12	198	11.1 ± 0.6	93.2 ± 4.4	11.5 ± 1.7
12–14	169	12.9 ± 0.6	96.8 ± 2.5	13.6 ± 1.5
14–20	42	14.9 ± 0.7	97.7 ± 2.5	14.5 ± 1.3

*n* absolute frequency of orthopantomograms, *M* mean value, *SD* standard deviation

Lateral cephalograms of 733 patients could be retrieved to analyse skeletal age according to the CVMS method. The allocation of lateral cephalograms and CVMS to the age groups (Table 5) shows that with increasing chronological age, CVMS, i.e. skeletal age, increased as well. Except for the group ≤ 10 years (*p* = 0.548), the CVMS were significantly different for boys and girls (*p* < 0.001/*p*^BH^ < 0.001).

**Table 5:** Distribution of lateral cephalograms (*n*) and cervical vertebrae maturation stage (CVMS) across age groups Verteilung der FRS(Fernröntgenseitenbild)-Aufnahmen (*n*) und der CVMS („Reifestadien der Halswirbel“) innerhalb der Altersgruppen

Age group	*n*	CVMS I *n* (%) 9.2 ± 1.8 years	CVMS II *n* (%) 10.7 ± 1.2 years	CVMS III *n* (%) 11.8 ± 1.2 years	CVMS IV *n* (%) 12.8 ± 1.2 years
Total	733	226 (30.8)	110 (15.0)	232 (31.7)	165 (22.5)
6–10	143	114 (79.7)	20 (14.0)	8 (5.6)	1 (0.7)
10–12	193	82 (42.5)	49 (25.4)	49 (25.4)	13 (6.7)
12–14	202	27 (13.4)	35 (17.3)	100 (49.5)	40 (19.8)
14–20	195	3 (1.5)	6 (3.1)	75 (38.5)	111 (56.9)

*n* absolute frequency of lateral cephalograms, *%* relative frequency of patients within an age group

Whereas no significant difference was evident for patients with skeletal class I, II and III in each chronological age group (*p* > 0.05), in children younger than 10 years (12 years without Bonferroni–Holm correction), the Demirjian point value was significantly different for skeletal classes I, II and III: skeletal class II patients had a higher Demirjian point value, i.e. an increased mineralisation stage, whereas skeletal class III patients had a smaller Demirjian point value than skeletal class I children in this age group (*p* = 0.008/*p*^BH^ = 0.040). A significant difference in dental age of patients with skeletal class I, II and III was only visible in children younger than 10 years, showing a higher dental age in class II and a lower dental age in class III than in class I patients (*p* = 0.032/*p*^BH^ = 0.160).

**Fig. 4:**
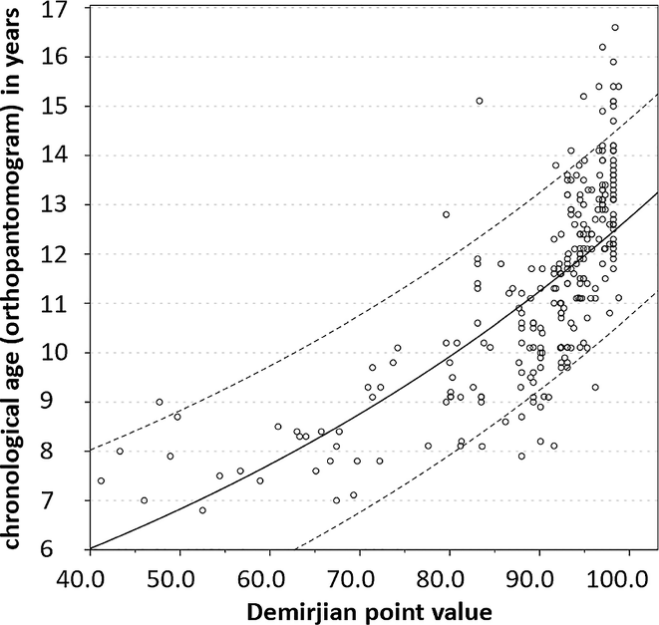
Regression model growth function (e-function) for boys to determine dental age in years based on Demirjian point value (range 0–100) Regressionsmodell Wachstumsfunktion (e-Funktion) für Jungen zur Bestimmung des dentalen Alters in Jahren basierend auf dem Demirjian-Punktwert (0–100)

**Fig. 5:**
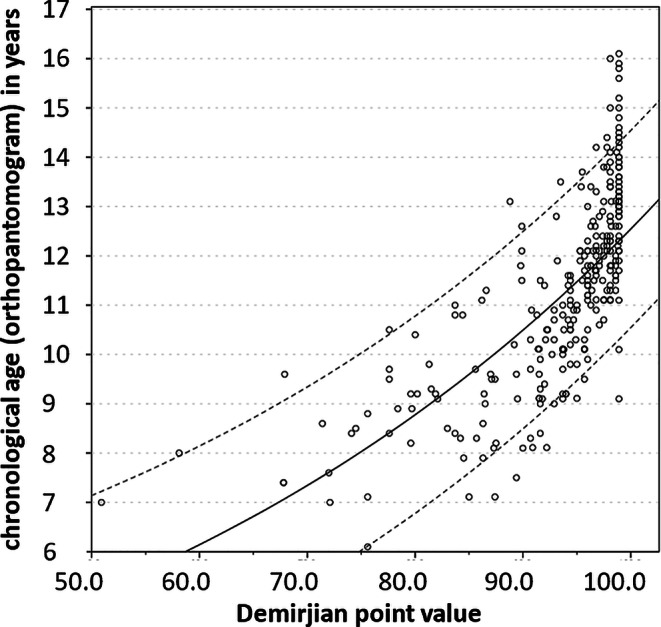
Regression model growth function (e-function) for girls to determine dental age in years based on the Demirjian point value (range 0–100) Regressionsmodell Wachstumsfunktion (e-Funktion) für Mädchen zur Bestimmung des dentalen Alters in Jahren basierend auf dem Demirjian-Punktwert (0–100)

Testing several regression models, the growth function had the best fit as it was revealed by the coefficient of determination R^2^ (R^2^_boys_ = 0.576, R^2^_girls_ = 0.546). Separated by gender, regression formulae were established to calculate dental age based on the Demirjian point value (Figs. 4 and 5):

2 $$\textit{Boys: dental age}=e^{1.297+0.012\times \textit{Demirjian point value}}$$

3 $$\textit{Girls: dental age}=e^{0.745+0.018\times \textit{Demirjian point value}}$$

These regression equations were used to express dental age based on the Demirjian point value in tables separated by gender. Chronological and dental age as determined by the new formulae were not significantly different (*p* = 0.131, paired two-tailed t‑test), thus confirming the validity of the new regression formulae for the Central-European population in question (Table 6).

**Table 6:** Reliability of the updated norm values for dental and skeletal age assessment Reliabilität der aktualisierten Normwerte für die dentale und die skelettale Altersbestimmung

*Age group*	*Age (years)*	*n*	*M*	*SD*	*t*	*Df*	*p*	*r*	*95% HL CI*
Total	Chronological	551	11.3	2.0	1.511	550	0.131	0.004	−0.21/0.02
	Dental (new)	551	11.2	1.3					
*Age group*	*Age (years)*	*n*	*M*	*SD*	*W*	*z*	*p*	*r*	*95% HL CI*
Total	Chronological	733	12.3	2.5	130051	0.113	0.910	0.004	−0.15/0.10
	Skeletal (new)	733	12.3	1.9					

*n* number orthopantomograms (dental)/lateral cephalograms (skeletal), *M* mean age in years, *SD* standard deviation, *t/W* test statistics paired two-tailed t‑/Wilcoxon rank sum test, *Df* degrees of freedom, *z* standard test statistics, *p* significance value, *r* effect size (Pearson correlation coefficient), *95% HL CI* 95% Hodges–Lehmann confidence interval

From 10 years onwards, all patients showed significantly different prevalences of CVMS in skeletal classes I, II and III: in the group 10–12 years (*p* = 0.006/*p*^BH^ = 0.024), significantly more class III patients and significantly less class II patients showed CVMS I than children with class I. CVMS II occurred less frequently in class III than in classes II and I. CVMS III and IV were reached significantly more often by class II and significantly less by class III patients than in class I children. In the age group 12–14 years (*p* = 0.027/*p*^BH^ = 0.054), CVMS III predominated and class II patients were the majority, followed by class I and finally class III patients. CVMS I was present mostly in class III patients, followed by class I and then class II children. CVMS II and IV showed similar prevalence in class I, II and III patients. In patients older than 14 years (*p* = 0.008/*p*^BH^ = 0.032) CVMS IV was significantly dominated by skeletal class III patients, followed by class II and finally class I patients. The opposite was true for CVMS III, which was found most often in class I patients, followed by class II and III patients. However, the effect size of this statistically significant finding was only small (0.1 < Cramer’s V < 0.3). In all chronological age groups, skeletal age according to Baccetti was significantly different from chronological age (*p* < 0.001/*p*^BH^ < 0.001): whereas children aged at least 10 years were younger according to skeletal age, the opposite was true for the age group < 10 years.

Updated conversion tables were established based on the evaluated Central-European population to determine skeletal age, separated by gender, based on the mean chronological age of 68% of the evaluated patients (±1 standard deviation of the mean; Table 7). As the statistically significant effect of skeletal class was only small, we did not distinguish between skeletal classes I, II and III in these tables. Compared to skeletal ages determined originally by Baccetti et al. [4], our population showed higher skeletal ages for all CVMS (Table 7). According to the Wilcoxon rank sum test, chronological and skeletal age were not significantly different, when our tables were applied (*p* = 0.910; Table 6).

**Table 7:** Cervical vertebrae maturation stage (CVMS)-based skeletal age assessment of a Central-European (present study) and South-European (Baccetti [4]) population CVMS(„Reifestadien der Halswirbel“)-basierte Bestimmung des skelettalen Alters einer mitteleuropäischen (vorliegende Studie) und einer südeuropäischen (Baccetti [4]) Population

		Skeletal age of our Central-European population					Skeletal age of Baccetti’s South-European population		
CVMS	Gender	*n*	M	±SD	Min	Max	*n*	M	±SD
I	Boys	130	10.3	1.8	6.8	15.8	30	9.2	1.8
	Girls	96	9.7	1.5	6.1	13.4			
II	Boys	63	12.0	1.5	9.1	15.2	30	10.7	1.2
	Girls	47	11.0	1.6	7.1	14.6			
III	Boys	131	13.8	1.7	9.4	18.2	30	11.8	1.2
	Girls	101	12.4	1.5	7.5	16.8			
IV	Boys	50	15.2	1.7	8.2	18.7	30	12.8	1.2
	Girls	115	14.6	2.0	10.1	19.4			

*n* number of patients, *M* mean chronological age, *SD* standard deviation in years (68% of patients), *Min* minimum, *Max* maximum

## Discussion

The aim of this retrospective study was to analyse the skeletal and dental age of a contemporary Central-European growing population using the methods of Baccetti et al. [4] and Demirjian et al. [12], and to investigate the impact of gender and skeletal class. Furthermore, we established updated norm values for dental and skeletal age according to these methods.

The study population was homogenous regarding the distribution of skeletal classes I, II and III, and gender for all age groups except for the group > 14 years, which was dominated by male patients. This can be explained by the exclusion of patients with a Demirjian point value of 100 or CVMS V, which is reached earlier by girls. Overall homogeneity ensured that gender- and dysgnathia-related differences were not caused by selection bias, but by statistical effects. Due to ethical reasons and the as low as reasonably achievable (ALARA) principle for applying radiation doses, patients were not radiographically examined longitudinally to evaluate skeletal and dental development. Instead, a retrospective, cross-sectional study design and appropriate statistics were used to establish norm values, generalisable for the population of interest.

In this study, gender-related differences were found for dental and skeletal age. As in the original publication of Demirjian et al. [12], the Demirjian point value was significantly higher for girls than for boys in the same chronological age group, showing the earlier mineralisation of teeth in female patients. However, in the final dental age determined by Demirjian et al., no significant differences were found between boys and girls because the conversion tables considered this observation already. Concerning skeletal age, girls revealed an accelerated development in almost all age groups as well. For example, between 10 and 12 years of age, CVMS I was present significantly more often in boys than in girls, whereas the opposite was true for CVMS III and IV. This phenomenon could be observed in the age groups 12–14 and > 14 years as well, illustrating that girls enter the pubertal growth spurt earlier and maintain this advancement until the end of growth. This difference was not evident for children younger than 10 years though, demonstrating that skeletal maturation does not start before the age of 10 years in both genders. Previous studies also found that girls start skeletal [7, 22, 31, 50, 59] and dental [12, 16, 27] maturation at a younger chronological age than boys. Since Baccetti et al. [4] did not analyse the influence of gender upon skeletal maturation, our results, incorporating this confounding factor, increase precision in the assessment of skeletal age.

According to our results, skeletal class seems to have a different influence on dental and skeletal age. In dental development, the only significant difference concerning skeletal class was observed in children below the age of 12 (Demirjian point value) or 10 years (dental age). Demirjian point values and dental age were higher in skeletal class II and lower in skeletal class III patients than in children presenting skeletal class I. Whereas overall our results indicate that dental age and Demirjian point value can be determined without differentiation of the skeletal classes, other authors reported an association between the sagittal jaw relation and dental development [32, 57]. Skeletal age, however, seems to be associated with the skeletal class. Whereas skeletal class II patients experienced growth spurt first, followed by class I patients, children with skeletal class III showed maximum growth latest, i.e. at the age of 14 years or older. Although this difference was statistically significant, the effect size was only small (0.1 < Cramer’s V < 0.3). Therefore, our results indicate that no additional effort in daily practice seems to be necessary to determine skeletal age considering the skeletal class. Further investigations are required to analyse the definite impact of sagittal dysgnathia on skeletal maturity.

Comparing dental and skeletal age of our study with the original publications of Demirjian et al. [12] and Baccetti et al. [4], respectively, differences to the original norm values are obvious. In most cases (< 14 years) of our recent population, dental age determined by Demirjian’s tables was significantly higher than the chronological age and would therefore result in an overestimation of patient age. A possible explanation for this difference might be the accelerated dental maturation of our contemporary Central-European population compared to Demirjian’s Canadian population from the 1970s. Such ethnically and population-based variances, when applying Demirjian’s method for dental age assessment were also described by other authors [1, 2, 8, 16, 27, 28, 34, 36, 56]. Therefore, we established updated norm values for dental age based on the mineralisation method of Demirjian et al. for a contemporary Central-European adolescent population using regression analysis as a growth function. Whereas skeletal class did not significantly affect dental age and therefore was neglected, gender was associated with dental development, leading to separate equations for boys and girls. The deviation from a norm value to be accepted was 2 years [43]. Applying the new formulae, reliability testing revealed no significant difference between mean chronological age and mean dental age, proving the validity of our equations.

Comparing mean chronological age and mean skeletal age as determined by Baccetti’s CVMS method, the latter was significantly higher. This shows that our investigated German patients had a delayed skeletal maturity compared to Baccetti’s Italian population, and that Central-European children seem to reach their pubertal growth spurt later than South-European adolescents. Therefore, new tables for skeletal age were established based on the Central-European population evaluated in this study to generate updated norm values without significant difference from the mean chronological age. Because of significant gender-related differences, the tables were separated for boys and girls. Since the clinical effect of skeletal class on skeletal age was small and to simplify the use of the tables in routine orthodontic diagnostics, subdivision for skeletal class I, II and III was not included in the new tables. Other authors, in contrast, did report an impact of skeletal class on skeletal age. Skeletal class III patients showed a longer pubertal growth peak starting at the same timepoint compared to skeletal class I children [25, 30].

Our findings are clinically relevant in several points. First, skeletal maturity assessed via cervical vertebrae maturation is significantly associated with mandibular growth potential [11, 14, 38, 44] and therefore influences treatment planning, when mandibular deviations or deficiencies are evident. Also orthodontic interventions in the upper jaw are affected by the remaining growth potential, as rapid maxillary expansion (RME) and combined RME/protraction are more effective before pubertal growth peak and at earlier stages of dental development, respectively [3, 15]. This could be explained by an association between skeletal maturity, assessed via cervical vertebrae maturation, and midpalatal suture maturity [35]. Dental age assessment is also clinically relevant for analysing facial growth, even if it does not allow identification of timing of the pubertal growth spurt because the latter is more associated with the development of cervical vertebrae [7, 39, 41, 50] and craniofacial growth [57]. Furthermore, dental age assessment might be easier to conduct, especially for general dentists having no lateral cephalogram devices, hence allowing a first examination. According to R^2^, the formulae explain 54.6% and 57.6% of variance in dental age of girls and boys, respectively. The remaining 45.4% and 42.4% of variance occur due to other reasons, including genetics, skeletal class, malnutrition and obesity [21, 42, 47]. Hence, even though dental and skeletal age assessment by the methods described reveal reliability, they should not be used solely, but as an addition to other clinical investigations including body height, onset of the menarche and signs and symptoms of disorders, each affecting physiological development. Finally, the choice of treatment devices depends on biological age: whereas skeletal maturity influences the success of functional orthopaedic appliances, dental development determines the time for insertion of fixed appliances.

A limitation of the applied method is radiographic imaging that might be imprecise due to anatomical, technical and rater-based reasons. To ensure a certain level of quality, radiographs of minor quality were excluded from the study. Furthermore, since radiographs from different treatment stages were included, some patients had already experienced functional orthopaedic therapies, which might have affected growth. As no longitudinal radiographic examination of a single individual patient was performed, actual craniofacial growth might have changed the skeletal class and hence affected the statistical analysis of the association between skeletal class and dental/skeletal age. However, our method to determine skeletal class, i.e. using the individualised ANB angle by Panagiotidis and Witt, considered already sagittal and vertical parameters, thereby increasing diagnostic precision. Another limiting factor is that the Demirjian method has no rule how to proceed in case of missing permanent teeth in the third quadrant. Although Demirjian point values appear to be precise, even if some teeth in the left quadrant are missing [33], it should be considered that tooth agenesis might influence rate of development of the neighbouring teeth [5]. Furthermore, premature loss of deciduous teeth was not taken into account. These might have eventually accelerated the eruption of permanent teeth, but not the rate of root formation [13]. Next, the CVMS method relies on the subjective analysis of an orthodontist. This could have introduced mistakes in the analysis of skeletal age. Also, Demirjian’s method is affected by some degree of subjective evaluation. However, inter- and intrarater reliability showed at least substantial measurement concordance and thus, guaranteed reliable measurements. Although the CVM stages allow a classification of remaining growth potential, the actual velocity of the peak mandibular growth cannot be predicted [6], introducing some uncertainty in treatment planning. Another limitation of this study is that the inclusion criteria did not ask for a certain, e.g. Caucasian, ethnicity. Thus, this fact was not evaluated as a confounder in the present study, although the German location of study places has probably ensured a mainly Caucasian population. The impact of ethnicity is controversially discussed in literature. Whereas some authors did not find a significant population- and ethnicity-related difference for skeletal and dental age assessment, others did [10, 26, 49]. Finally, according to the inclusion criteria, only patients without any systemic diseases were considered, so that our results can be generalised with certainty only to generally healthy patients.

## Conclusions

Using a regression formula based on the mineralisation stages of the left mandibular teeth and conversion tables for CVM stages, we established new norm values to assess dental and skeletal age according to the methods by Demirjian et al. and Baccetti et al., respectively. The new values, updated for a contemporary Central-European growing population and separated by gender, might improve orthodontic diagnosis and be helpful in planning orthodontic treatment. Further investigations should be conducted to test the clinical relevance of the association between the skeletal class and skeletal age.
